# Low Genetic Diversity of *Plasmodium vivax* Circumsporozoite Surface Protein in Clinical Isolates from Southern Thailand

**DOI:** 10.3390/tropicalmed9050094

**Published:** 2024-04-24

**Authors:** Tachin Khulmanee, Thanyapit Thita, Kanyanan Kritsiriwutinan, Usa Boonyuen, Aminoh Saai, Kanjana Inkabjan, Rimi Chakrabarti, Pradipsinh K. Rathod, Srivicha Krudsood, Mathirut Mungthin, Rapatbhorn Patrapuvich

**Affiliations:** 1 Drug Research Unit for Malaria, Faculty of Tropical Medicine, Mahidol University, Bangkok 10400, Thailand; 2 Faculty of Medical Technology, Rangsit University, Pathum Thani 12000, Thailand; 3 Department of Molecular Tropical Medicine and Genetics, Faculty of Tropical Medicine, Mahidol University, Bangkok 10400, Thailand; 4 Bannang Sata Hospital, Yala 95130, Thailand; 5 Suanphueng Hospital, Ratchaburi 70180, Thailand; 6 Department of Chemistry, University of Washington, Seattle, WA 98195, USA; 7 Clinical Malaria Research Unit, Faculty of Tropical Medicine, Mahidol University, Bangkok 10400, Thailand; 8 Department of Parasitology, Phramongkutklao College of Medicine, Bangkok 10400, Thailand

**Keywords:** malaria, *Plasmodium vivax*, circumsporozoite surface protein, Thailand

## Abstract

The genetic diversity within the circumsporozoite surface protein (PvCSP) of *Plasmodium vivax*, the predominant malaria species in Thailand, is primarily observed in the northwestern region along the Thailand–Myanmar border. However, as *P. vivax* cases shift to southern provinces, particularly Yala Province near the Thailand–Malaysia border, PvCSP diversity remains understudied. Between 2018 and 2020, 89 *P. vivax* isolates were collected in Yala Province, a significant malaria hotspot. Employing polymerase chain reaction amplification, restriction fragment length polymorphism (PCR-RFLP), and DNA sequencing, the gene encoding PvCSP (*Pvcsp*) was analyzed. All Yala *P. vivax* isolates belonged to the VK210 type, distinct from strains in the western region near the Myanmar border. The central repeat region of *Pvcsp* revealed two common peptide repeat motifs—GDRADGQPA and GDRAAGQPA—across all southern isolates. Sequence analysis identified two subtypes, with S1 more prevalent (92%) than S2 (8%). This study underscores the limited diversity of VK210 variants of *P. vivax* populations in southern Thailand. These baseline findings facilitate monitoring for potential new parasite variants, aiding in the future control and management of *P. vivax* in the region.

## 1. Introduction

Malaria continues to be a global infectious disease of significance. Among the six human-infecting *Plasmodium* species (*Plasmodium falciparum*, *P. vivax*, *P. malariae*, *P. ovale curtisi*, *P. ovale wallikeri*, and *P. knowlesi*), *P. vivax* stands out as the primary cause of most cases of malaria-related morbidity and mortality in regions where malaria is endemic, excluding Africa [1]. *P. vivax* presents a greater challenge to control efforts compared to *P. falciparum* because of its ability to hide in a dormant form, known as a hypnozoite, in the human liver. This dormant form can reactivate weeks or months after the primary infection, leading to relapses [2,3]. While *P. vivax* has historically been associated with benign malarial infections, there is increasing evidence of severe complications in some *P. vivax*-infected patients [4,5]. Moreover, the emergence and spread of drug-resistant strains, particularly in the Thai-Myanmar area, further contribute to the burden caused by the *P. vivax* parasites [6,7,8,9].

As *P. falciparum* cases declined in Thailand following the initiation of the Mekong Malaria Elimination (MME) program in 2012 [1], *P. vivax* is emerging as the dominant human malaria species and now constitutes 96% of all malaria cases in the country [10]. Malaria transmission in Thailand is concentrated in three main hotspots: the northeastern border with Lao PDR and Cambodia (particularly Ubon Ratchathani and Srisaket provinces), the western border with Myanmar (especially Tak Province), and the southern border adjacent to Malaysia (particularly Yala Province) [11]. To apply effective interventions to the goal of eliminating malaria in Thailand by 2024 [12], it is important to study the genetic diversity of *P. vivax* populations recently circulating in these hotspots.

The gene encoding the circumsporozoite surface protein of *P. vivax* (*Pvcsp*) is highly polymorphic and serves as a valuable genetic marker for analyzing diversity within *P. vivax* populations. The circumsporozoite surface protein (CSP) plays a crucial role in sporozoite maturation, mosquito salivary gland invasion, and hepatocyte invasion [13,14], making it a central focus in malaria vaccine development. CSP-based vaccines have shown promise in clinical trials, preventing sporozoites from infecting the liver [15]. However, a noteworthy concern persists regarding the genetic diversity of *Pvcsp* within the natural *P. vivax* population. CSP consists of a highly polymorphic central repeat region (CRR) flanked by two conserved domains: the pre-repeat N-terminal region and the post-repeat C-terminal region. Two major *P. vivax* variants, known as VK210 and VK247, are distinguished by the distinct peptide repeat motifs (PRMs) in the CRR: GDRA [D/A] GQPA in VK210 and ANGAGNQPG in VK247 [16,17]. Both VK210 and VK247 variants are prevalent globally. The VK210 strain has been reported to be the predominant type in numerous countries, including Thailand [18,19,20,21,22,23,24,25,26], while VK247 has been identified as a common type in some endemic areas of Latin America [27]. VK210 displays higher genetic diversity compared to VK247 [28,29]. Genetic variations in the N-terminal non-repeat region of *Pvcsp* are limited, while in the C-terminal region, different patterns of octapeptide insertion (ANKKAEDA in VK210) and tetrapeptide repeat (GGNA) motifs have been identified. In addition, a *P. vivax*-like variant containing APGANQ (E/G)GAA motifs in the CRR is also present in malaria-endemic regions of Papua New Guinea, Brazil, Indonesia, and Madagascar [30,31].

The majority of data concerning *Pvcsp* diversity in Thailand have been gathered from the northwestern region along the Myanmar border [19,32,33,34]. Genetically distinct populations of *P. vivax* have been identified in malaria transmission hotspots in Thailand: Tak in the west and Ubon Ratchathani in the northeast [35]. Between 2015 and 2019, the number of *P. vivax* cases in Thailand decreased by approximately 68%, from 14,252 to 4860 [36]. Since 2017, the epicenters of *P. vivax* cases have shifted to the southern provinces, especially Yala. In 2015, Yala accounted for 5.8% (831 out of 14,252) of the country’s *P. vivax* cases, which increased to 28% (1367 out of 4860) in 2019 [36]. Interestingly, the genetic nature of *Pvcsp* within the *P. vivax* population in Yala has not been thoroughly studied. Therefore, the present work examined the diversity of *Pvcsp* in *P. vivax* isolates collected from Yala Province in southern Thailand using nested PCR—restriction fragment length polymorphism (RFLP) and DNA sequencing. These findings will provide essential information for the surveillance and management of *P. vivax* malaria.

## 2. Materials and Methods

### 2.1. Sample Collection

**Figure 1:**
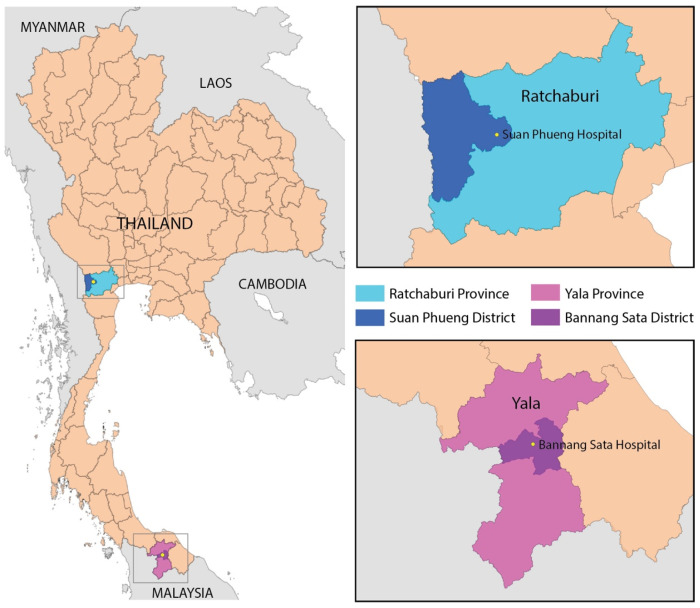
A map of Thailand illustrating the locations of the study sites. Blood samples were collected from *P. vivax*-infected patients residing in Bannang Sata District, Yala Province, and the Suan Phueng District, Ratchaburi Province.

Between April 2018 and June 2020, 95 blood samples that tested positive for malaria infection were collected in Bannang Sata Hospital in Yala Province, near the Thai-Malaysian border (Figure 1). These samples were placed in a tube with ethylenediaminetetraacetic acid (EDTA), and *Plasmodium* infection was diagnosed through microscopic analysis of Giemsa-stained thin blood smears. One-milliliter blood samples were cryopreserved at a 1:2 ratio with Glycerolyte 57 solution (Baxter, Deerfield, IL, USA) and stored at −20 °C before transportation under dry ice to the Drug Research Unit for Malaria (DRUM) at Mahidol University in Bangkok, Thailand. At DRUM, the samples were stored at −80 °C until DNA extraction was performed on later days. Following the COVID-19 pandemic, isolates from 10 frozen *P. vivax*-infected samples from Suanphueng Hospital in Ratchaburi Province (Figure 1), which were collected between December 2021 and May 2022, served as representatives of *P. vivax* from along the Thai-Myanmar border in the west. All laboratory work in this study was conducted at DRUM. Sample collection was approved by the Human Ethics Committee of the Faculty of Tropical Medicine, Mahidol University (approval number: MUTM 2018-031-05).

### 2.2. DNA Template Preparation

Genomic DNA (gDNA) was extracted from blood samples using the QIAamp DNA Blood Mini kit, following the manufacturer’s instructions (QIAGEN, Hilden, Germany). Briefly, DNA was isolated from a 500 μL blood sample. The extracted DNA was eluted in AE buffer (10 mM Tris-HCL; 0.5 mM EDTA; pH 9.0) to a final volume of 100 μL. The DNA concentration in the eluant was determined using a NanoDrop 2000 spectrophotometer (Thermo Fisher Scientific, Waltham, MA, USA). Confirmation of the microscopic diagnosis of *Plasmodium* species was conducted using PCR-based protocols [37].

### 2.3. Genotyping of Pvcsp by Polymerase Chain Reaction-Restriction Fragment Length Polymorphism (PCR-RFLP)

*Pvcsp* genotyping of *P. vivax*-positive samples was conducted using a PCR-RFLP analysis, following a previously established protocol [34] with some modifications. Briefly, the *Pvcsp* gene was first amplified using a nested PCR assay. The primers used were VCS-OF (ATGTAGATCTGTCCAAGGCCATAAA) and VCS-OR (TAATTGAATAATGCTAGGACTAACAATATG) as primary primers, with VCS-NF (GCAGAACCAAAAAATCCACGTGAAAATAAG) and VCS-NR (CCAACGGTAGCTCTAACTTTATCTAGGTAT) serving as nested primers. The amplification reaction was carried out in a total volume of 25 μL, containing 12.5 μL of PCR Green Master Mix (Promega, Madison, WI, USA), 10 μM of each primer, and 1 μL of gDNA extracted from the blood samples. For the primary PCR reaction, the cycling conditions were as follows: initial denaturation at 95 °C for 5 min, followed by 25 cycles of denaturation at 94 °C for 1 min, annealing at 56 °C for 90 s, and extension at 72 °C for 2 min, and a final extension at 72 °C for 5 min. A second round of amplification was performed in a total volume of 25 μL using 1 μL of PCR products from the first amplification as a template. The PCR conditions for the second reaction were an initial denaturation at 95 °C for 5 min, followed by 30 cycles of denaturation at 94 °C for 1 min, annealing at 62 °C for 90 s, extension at 72 °C for 2 min, and final extension at 72 °C for 5 min using a Mastercycler ProS vapo.protect device (Eppendorf, Hamburg, Germany). Subsequently, 10 μL of the nested PCR products were individually digested with restriction enzymes *Alu*I and *Bst*NI (New England Biolabs Inc., Hitchin, UK) for 4 h in a total volume of 30 μL according to the manufacturer’s specifications. The resulting DNA fragments were visualized with 1.5% agarose gel electrophoresis and SYBR-safe DNA Gel Stain (Invitrogen, Waltham, MA, USA) and imaged with a Bio-Rad Gel Doc XR+ imaging system (Bio-Rad, Hercules, CA, USA).

### 2.4. Analysis of Pvcsp Gene Sequence

DNA sequence analysis was conducted on *Pvcsp* DNA to identify polymorphisms in its CRR, pre-repeat, and post-repeat regions. The nested PCR products of *Pvcsp* were sequenced using the ABI 3730XL sequence analyzer (Macrogen, Seoul, Republic of Korea). Subsequently, the resulting electropherograms were processed and assembled using BioEdit software, version 7.2.5 [38]. Nucleotide and amino acid sequences were aligned and compared to published sequences (GenBank access numbers: M28746 for VK210 and M28745 for VK247) using the ClustalW alignment method. A phylogenetic tree was constructed from the aligned nucleotide sequences and from deduced amino acid sequences using the neighbor-joining method of MEGA version 11 [39]. This facilitated an understanding of the genetic relationships between regional *P. vivax* isolates. The nucleotide sequences of *Pvcsp* analyzed in this study have been submitted to the GENBANK database under the accession numbers PP084327-PP084424.

## 3. Results

A total of 95 blood samples collected between 2018 and 2020 from malaria patients in Yala Province were examined for *P. vivax* infection using PCR and microscopy. Among the 95 samples, 88 were single-infected with *P. vivax*, 6 were with *P. falciparum,* and 1 harbored both species. *P. vivax* infection was observed in 94% (89/95) of the collected samples. These *P. vivax* isolates were equally distributed between male (54%) and female (46%) patients, with a mean age (mean ± SD) of 34.7 ± 18.5 years (range 4–90). All *P. vivax* isolates, including those from mixed infection cases, were used for the analysis of polymorphisms in the *Pvcsp* gene.

### 3.1. Genotyping of Pvcsp

**Figure 2:**
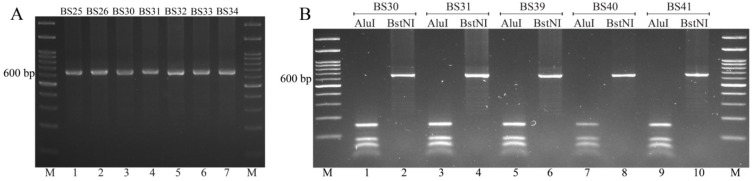
PCR product sizes and genotypes of *Pvcsp*. (**A**) PCR products of *Pvcsp* from representative southern isolates (BS25, BS26, BS30, BS31, BS32, BS33, and BS34) from Yala Province. (**B**) RFLP analysis of *Pvcsp* fragments. In odd-numbered lanes, the PCR products were digested with *Alu*I (an enzyme that cuts repeatedly in the VK210 repeat region). In even-numbered lanes, the fragments were digested with *Bst*NI (an enzyme that cuts repeatedly in the VK247 repeat region). Paired digestions of the fragments obtained from representative southern isolates (BS30, BS31, BS39, BS40, and BS41) from Yala Province are presented. A 100 bp DNA ladder was used as a molecular weight marker (M).

Amplification of *Pvcsp* fragments was successful for 88 isolates (99%, 88/89). Despite repeated attempts, one isolate could not be amplified. The PCR products displayed limited size variants ranging from 600 bp to 650 bp (Figure 2A). *Pvcsp* was further genotyped using RFLP to distinguish between the two peptide repeat types, VK210 and VK247. All Yala isolates belonged to the VK210 variant (Figure 2B).

**Figure 3:**
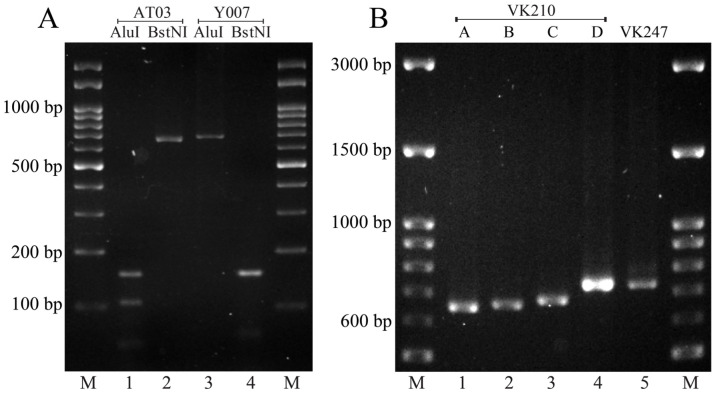
(**A**) PCR-RFLP analysis of *Pvcsp* fragments from western samples in Thailand. The fragments were digested by *Alu*I and *Bst*NI, which degrade the VK210 and VK247 types, respectively. Paired digestions of the fragments obtained from representative isolates (AT03 for VK210 and Y007 for VK247) are presented. (**B**) Fragment size ranges for *Pvcsp* gene from western samples. Four VK210 allelic variants were defined according to size (lanes 1–4): A = 600 to <650 bp, B = ~650 bp, C ≥ 650 to 700 bp, and D = ~700 bp. The fragment size of a representative VK247 is presented in lane 5. A 100-bp DNA ladder was used as the molecular weight marker (M).

In the western samples, both VK210 (90%, 9/10) and VK247 (10%, 1/10) were detected (Figure 3A). On the basis of PCR band size variations, four distinguishable size variants with lengths of between 600 bp and 700 bp were observed among the western VK210 strains (Figure 3B). Allelic size variant C (>650–700 bp) dominated at a frequency of 56% (5/9).

### 3.2. Diversity of Pvcsp Gene

Direct sequencing was conducted on amplified *Pvcsp* of each *P. vivax* isolate. No variation was observed in the N-terminal non-repeat region. In the CRR, only two PRMs—GDRADGQPA and GDRAAGQPA—were abundant and widely distributed across all southern isolates. These PRMs were repeated 18 times in all isolates. Additionally, the PRMs GNGAGGQAA and GDGAGGQAA were identified as the last repeat units in the CRR, categorizing the southern isolates into S1 and S2 subtypes (Table 1). S1 accounted for 92% (81/88) of the *P. vivax* VK210 variants in Yala. No insertion sequence with the octapeptides ANKKAEDA was observed following the CRR, and the GGNA unit after the CRR was repeated three times in all cases. In contrast, western isolates displayed not only the two common PRMs (GDRADGQPA and GDRAAGQPA) but also GGRADGQPA. The frequency of PRM repetition ranged from 18 to 19 times. The PRM GNGAGGQAA was consistently identified as the last repeat unit in the CRR among all western isolates. Furthermore, variations were found in the post-repeat regions of the western samples. An octapeptide insertion was observed, with the GGNA unit appearing after the CRR, repeated between 2 and 10 times. The western VK210 variants were categorized into W1–W5 subtypes based on variations in the order and frequency of the PRM repeats, as listed in Table 1, with W4 being the most prevalent, at 56% (5/9).

**Figure 4:**
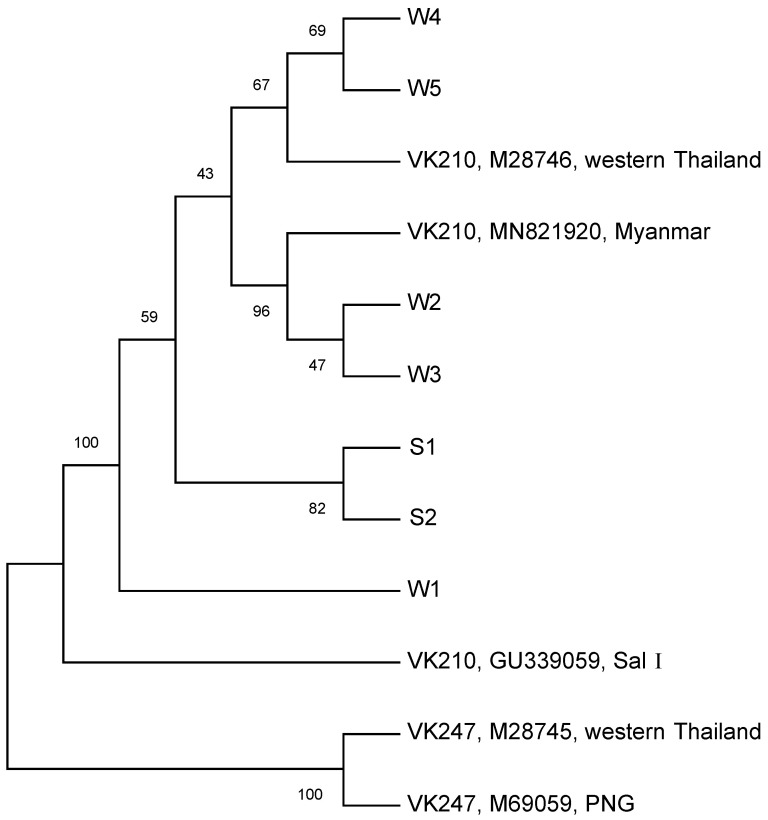
Phylogenetic relationships among the *P. vivax* allelic variants. The nucleotide acid sequences of *Pvcsp* fragments from all identified VK210 subtypes were compared with the published sequences of reference VK210 and VK247 strains. The bootstrap values are shown on the branches and indicate the number of times out of 1000 replications.

The *Pvcsp* sequences of the VK210 subtypes (S1 and S2 from Yala and W1–W5 from Ratchaburi) were compared with the reference sequences available in GenBank (M28746 for VK210 from Thailand, GU339059 for VK210 strain Salvador I, MN821920 for VK210 from Myanmar, M28745 for VK247 from Thailand, and M69059 for VK247 from Papua New Guinea). Both the VK210 and VK247 type sequences are clustered into two distinct divisions (Figure 4). For the VK210 type, each division consisted of several sub-divisions. The phylogenetic tree illustrates the separation between the southern *P. vivax* variants and those from the western region. W4 and W5 were closer in proximity to the reference VK210 from western Thailand collected in 1989 [17] than the other subtypes. W2 and W3 were closely related to *P. vivax*, which was collected from Naung Cho, Myanmar, in 2013 [28]. W1 had an additional repeat motif, GGRADGQPA, distinct from the common GDRA [D/A] GQPA in the CRR, setting it apart from the other variants analyzed in this study.

### 3.3. Prevalence of the VK210 Variants

**Figure 5:**
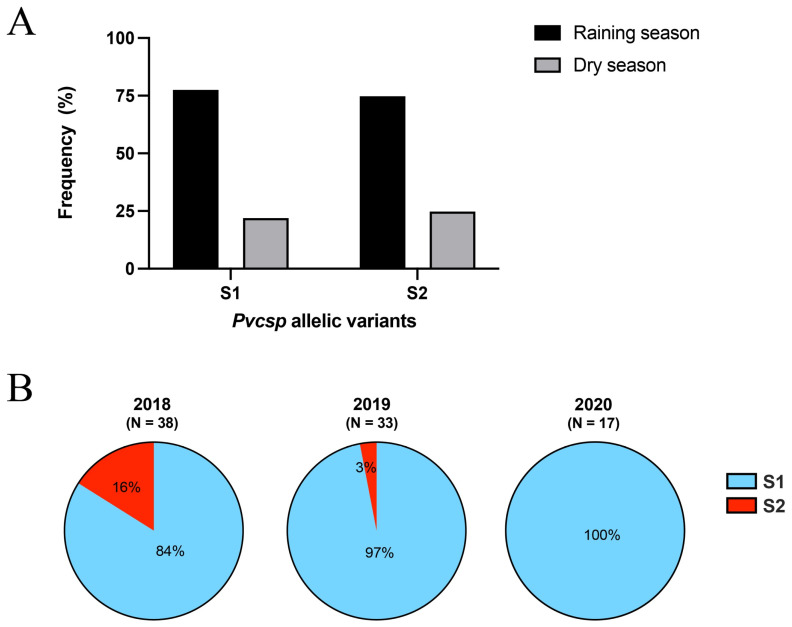
(**A**) Distribution of VK210 variants during different seasons: June to October (rainy season) and November to May (dry season). (**B**) Frequency distribution of *Pvcsp* allelic variants, S1 and S2, in southern *P. vivax* isolates. Analysis of 88 isolates from the period from 2018 to 2020.

S1 was the predominant subtype of *P. vivax* VK210 in Yala (Table 1). A comparison of S1 and S2 variant frequencies between the rainy season (June to October) and the dry season (November to May) was conducted to assess potential seasonal fluctuations. Both S1 and S2 variants were more prevalent during the rainy season (~75%) than the dry season (~25%) (Figure 5A). Infection with the S2 variant was less frequent and declined over the course of the study, from 2018 to 2020, with only S1 being present in 2020 (Figure 5B). Notably, in 2020, the collection period spanned only 6 months, from January to June. In the western isolates, W4 accounted for 56% of the *P. vivax* VK210 variants, while the other four variants represented 11% (1/9) each (Table 1). Because western isolates were exclusively collected during the dry season, the presence of seasonal fluctuations in the western allelic variants could not be assessed.

## 4. Discussion

Despite the significant reduction in malaria transmission in Thailand over the past decade, high genetic diversity among malaria parasites remains prevalent, especially in western provinces. Yala is a major malaria-endemic province on the Thai-Malaysian border, but the diversity of the malarial parasites circulating in this region remains poorly understood. This is the first report on *Pvcsp* diversity in southern Thailand, and the findings indicate that all *P. vivax* isolates from Yala belonged exclusively to the VK210 variant, whereas both VK210 and VK247 variants coexisted in the western region. The VK210 variant dominated, representing around 90% of western isolates. This aligns with previous studies that identified VK210 as being the most prevalent variant in global and Thai *P. vivax* populations [19,20,22,28,32,40,41,42,43,44].

The translated nucleotide sequences of *Pvcsp* in the samples from both southern and western regions revealed the presence of repetitive 9-mer PRMs that were repeated 18–19 times, a common occurrence in most malaria-endemic areas worldwide [45]. Analyzing the arrangement of PRM units in the CRR and investigating sequence-length polymorphism between samples from the two regions showed the presence of only two distinct allelic types among the 88 Yala isolates, whereas the 10 Ratchaburi isolates included five different allelic types. This suggests the *Pvcsp* gene has a comparatively low level of diversity within the southern region. Furthermore, the allelic types of the VK210 variants in the south differed from those in the western isolates, confirming the limited parasite connectivity among the major malaria hotspots in Thailand [11,35]. These differences between the western and southern variants may serve as indicators for tracking parasites from different regions.

The cell adhesive motif (KLKQP) in the N-terminal non-repeat region, exposed by proteolytic cleavage after the interaction between sporozoite and hepatocyte [14], was conserved among all VK210 and VK247 variants analyzed in this study. At the C-terminal region, various polymorphic characteristics were observed in the western VK210 variants, including the insertion sequence of octapeptides and the frequency of the GGNA repeat. Among the five VK210 subtypes, W2 and W3 displayed the ANKKAEDA insertion, similar to isolates from Myanmar [45]. This suggests a potential common origin and dissemination of these parasites by travelers across the Thai–Myanmar border. GGNA repeats are typically observed as singular occurrences in isolates from South Asia, West Asia, and Africa [45]. However, in contrast, the majority of isolates from both the southern and western regions of Thailand in this study displayed variable GGNA repeat frequencies, with the motif occurring either twice or more than three times. This pattern aligns with observations in isolates from Southeast Asia and the China–Myanmar border area [45]. Notably, W5 variants exhibited as many as 10 GGNA repeats. The VK210 variants identified in the present western isolates differed from the reference western 1989 isolate [17], suggesting that the prevalent genotype has changed. The relatively higher diversity observed in the western isolates could be attributed to cross-border exchanges [46] that may have led to the introduction and establishment of novel parasite genotypes in the western region. Additionally, a recent report indicated that *Pvcsp* from Myanmar had more genetic polymorphisms than those from other countries [28].

The genetic diversity of southern *Pvcsp* was notably lower compared to that reported in the western part of Thailand. In Yala samples, this study identified two VK210 variants, S1 and S2, with a decline in S2 over time, resulting in the predominance of S1 in 2020. The prevalence of *P. vivax* variants in endemic areas may be influenced by the distribution of *Anopheles* mosquito species [18,47,48]. The decrease in S2 could be associated with the varying susceptibility of mosquito vectors to *P. vivax* in the region, emphasizing the need for entomological studies for effective malaria control. In Thailand, a minimum of nine *Anopheles* species, including *An. minimus*, *An. maculatus*, *An. dirus*, *An. sawadwongporni*, *An. karwari*, *An. barbirostris*, *An. campestris*, *An. hodgkini Reid*, and *An. annularis*, have been identified as potential *P. vivax* malaria vectors [49,50,51,52]. The primary malaria vectors in both western and southern Thailand are *An. minimus*, *An. maculatus*, and *An. dirus* [49]. Thus, the genetic differentiation observed between the Yala and Ratchaburi isolates may be influenced by additional factors such as host immune pressure and the migration of people within the endemic regions.

The initial discovery of the VK247 variant in Thailand [17] prompts questions about why it is not found in other parts of the country. In Yala, the VK210 subtype was dominant, with VK247 notably missing during the observation period. This lower occurrence might be due to variations in how VK247 develops in local mosquito populations or another hypothesis focusing on immune responses [53]. The presence of antibodies targeting the VK247 subtype could limit its sporozoite production, reducing its prevalence in mosquitoes. This, in turn, could impact the parasite load in infected individuals in the region.

The identification of low genetic diversity, as indicated by other genetic markers such as microsatellites [54] and merozoite surface protein 3-alpha (*PvMSP-3α*) [55], has also been reported for *P. vivax* isolates from Yala. The province’s geographical isolation, distinct occupation, and culture may contribute to its unique characteristics. The southern Muslims, often employed by rubber plantations, have a lifestyle different from those of other ethnic groups in the country. Malaria cases are predominantly indigenous and tend to be concentrated in the areas surrounding rubber plantations. Moreover, the restriction of migration to Yala is, in part, influenced by the ongoing regional violence in Thailand’s southernmost provinces: Yala, Narathiwat, and Pattani. This constrained human mobility potentially resulted in the clonal characteristic of *P. vivax* isolates in Yala. The scarce occurrence of *Pvcsp* variants indicates that a PvCSP-based vaccine might exhibit higher efficacy in Yala compared to the western provinces.

Cross-border migration could influence the genetic diversity of parasite populations. Yala shares borders with the states of Kedah and Perak in Peninsular Malaysia. Southern Thailand and Peninsular Malaysia have similar malaria vectors, with *An. maculatus* playing a significant role in transmitting human malaria along the Thai-Malaysian border [49,56]. However, several factors hinder the genetic exchange between Yala and Malaysian isolates. These include low malaria transmission rates in neighboring Malaysian states, where the incident rates are below 0.1 per 1000 [57], and a significantly reduced volume of cross-border travel in this region, mainly due to political unrest in southern Thailand. Moreover, Malaysia has reported zero indigenous human malaria cases since 2018 [58], further reducing the cross-border impact on *P. vivax* diversity in Yala.

Genetic variation can significantly impact the traits, treatment responses, and other biological characteristics of the parasite. Relapses play a significant role in promoting *P. vivax* transmission and sustaining local diversity. Given that a substantial proportion of clinically observed *P. vivax* infections result from relapses [59,60], the reliability of the *Pvcsp* marker as a hypnozoite-specific trait is under consideration. There is evidence indicating that VK247 sporozoites produce a greater number of hypnozoites compared to VK210 sporozoites [61,62]. However, information on the correlations between *P. vivax* sub-variants, specifically the VK210 or VK247 types, and hypnozoite frequencies and relapse rates is currently lacking and requires further investigation. A challenge faced by those treating *P. vivax* is the potential for diverse drug responses based on the parasite genotype. A published report indicates that resistance profiles to chloroquine and mefloquine are not correlated with the VK210 subtypes of *P. vivax* isolates from the Amazon region in Brazil [63]. Chloroquine plus primaquine remains effective for treating uncomplicated vivax malaria in Thailand, with a cure rate exceeding 95% [64]. In contrast to *P. vivax* isolates from other malaria hotspots, Yala isolates not only display lower genetic diversity but also demonstrate higher sensitivity to all currently available antimalarial drugs [65]. *P. vivax* isolates from the Myanmar and Cambodia borders, with a parasite clearance time (PCT) longer than 24 h after chloroquine treatment, exhibit higher diversity compared to those from Yala, where the PCT is less than 24 h [54]. A study on the molecular detection of drug-resistant malaria in southern Thailand further affirmed the sustained effectiveness of chloroquine treatment in Yala [66]. The findings underscored the distinct genetics of Yala isolates compared with those in other malaria hotspots.

Consequently, understanding parasite population genetics could play a crucial role in designing and monitoring strategies for the elimination of the parasite. In this study, *P. vivax* isolates were collected from a single province in southern Thailand, which restricts the results’ ability to represent the region-wide genetic diversity of *Pvcsp*. To overcome this limitation, a more extensive study involving a large number of *P. vivax* isolates from diverse endemic sites in the southern region is required. The study acknowledges the importance of comparing genetic diversity in *P. vivax* isolates from both humans and vectors. However, due to the unavailability of vector-derived isolates in the study region, this comparative analysis could not be conducted. As a result, drawing definitive conclusions and assessing the finding’s relevance for vaccine control strategies were hindered. Despite this setback, the significance of such comparisons is recognized and will be addressed in future research efforts.

## 5. Conclusions

The current study aimed to investigate the genetic diversity of *Pvcsp* in clinical isolates from southern Thailand, specifically those from near the Thai–Malaysia border. An analysis of 88 sequences of *Pvcsp* from a single endemic area, Yala Province, unveiled substantially lower genetic diversity compared to samples from the western region. The conserved genetic characteristics of *P. vivax* in the south, distinct from those observed in the west, imply that control and elimination strategies may have to be independently tailored to each endemic area.

## Figures and Tables

**Table 1:** Distribution of peptide repeat motifs (PRMs) in the central repeat region (CRR) of *Pvcsp* (VK210) variants.

Types	Amino Acid Sequences in CRR	Repeat No.	Insertion of ANKKAEDA	No. of GGNA Repeat	No. of Cases
S1	○○■○○■○■○■■○■○■■■◇	18	No	3	81
S2	○○■○○■○■○■■○■○■■■◆	18	No	3	7
Ref_W1989 *	○○○■○■○■○■■○■○■■■◇	18	No	3	
W1	∆○○■○■■○■○■○○■○■■◇	18	No	3	1
W2	○○○■○■○■○○○■■○■■■◇	18	Yes	2	1
W3	○○○■○■○■○■○■■○■■■◇	18	Yes	2	1
W4	○○○■○■○■○■■■○■○■■■◇	19	No	3	5
W5	○○○■○■○■○■■○■○■■■◇	18	No	10	1

Note: GDRADGQPA—○, GDRAAGQPA—■, GGRADGQPA—∆, GNGAGGQAA—◇, GDGAGGQAA—◆. * Reference VK210 *Pvcsp* sequence with GenBank access number M28746.

## Data Availability

Data will be made available upon request.
